# 170. Reduction in Bloodborne Pathogen Splash Exposures After Implementation of Universal Masking and Eye Protection for COVID-19

**DOI:** 10.1093/ofid/ofab466.170

**Published:** 2021-12-04

**Authors:** Marci Drees, Tabe Mase, Jennifer Garvin, Kimberly Miller

**Affiliations:** 1 ChristianaCare, Newark, DE; 2 Christiana Care Health System, Newark, DE

## Abstract

**Background:**

While splashes to the eyes, nose and mouth can often be prevented through appropriate personal protective equipment (PPE) use, they continue to occur frequently when PPE is not used consistently. Due to the COVID-19 pandemic, we implemented universal masking and eye protection for all healthcare personnel (HCP) performing direct patient care and observed a subsequent decline in bloodborne pathogen (BBP) splash exposures.

**Methods:**

Our healthcare system, employing >12,000 healthcare personnel (HCP), implemented universal masking in April 2020 and eye protection in June 2020. We required HCP to mask at all times, and use a face shield, safety glasses or goggles when providing direct patient care. Occupational Safety tracked all BBP exposures due to splashes to the eyes, nose, mouth and/or face, and compared exposures during 2020 to those in 2019. We estimated costs, including patient and HCP testing, related to splash exposures, as well as the additional cost of PPE incurred.

**Results:**

In 2019, HCP reported 90 splashes, of which 57 (63%) were to the eyes. In 2020, splashes decreased by 54% to 47 (36 [77%] to eyes). In both years, nurses were the most commonly affected HCP type (62% and 72%, respectively, of all exposures). Physicians (including residents) had the greatest decrease in 2020 (10 vs. 1 splash exposures [90%]), while nurses had a 39% decrease (56 vs. 34 exposures). Nearly all of the most common scenarios leading to splash exposures declined in 2020 (Table). We estimated the cost of each BBP exposure as &2,940; this equates to a savings of &123,228. During 2020, we purchased 65,650 face shields, safety glasses and goggles (compared to 5303 similar items in 2019), for an additional cost of &238,440.

Specific activities identified as leading to bloodborne pathogen splash exposures, 2019 vs. 2020.

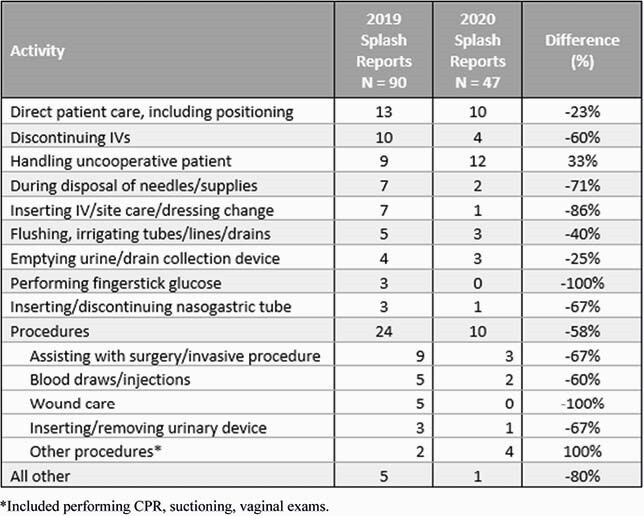

**Conclusion:**

We observed a significant decline in splash-related BBP exposures after implementing universal masking and eye protection for the COVID-19 pandemic. While cost savings were not observed, we were unable to incorporate the avoided pain and emotional trauma for the patient, exposed HCP, and coworkers. This unintended but positive consequence of the COVID-19 pandemic exemplifies the need for broader use of PPE, particularly masks and eyewear, for all patient care scenarios where splashes may occur.

**Disclosures:**

**All Authors**: No reported disclosures

